# Midwives’ Knowledge, Attitudes, and Practice Regarding COVID-19 Vaccination for Pregnant Women: A Nationwide Web-Based Survey in Italy

**DOI:** 10.3390/vaccines11020222

**Published:** 2023-01-19

**Authors:** Grazia Miraglia del Giudice, Giorgia Della Polla, Lucio Folcarelli, Annalisa Napoli, Raffaella Punzo, Martina Peracchini, Italo Francesco Angelillo

**Affiliations:** 1 Department of Experimental Medicine, University of Campania “Luigi Vanvitelli”, Via Luciano Armanni 5, 80138 Naples, Italy; 2 Department of Public Health and Laboratory Services, Teaching Hospital of the University of Campania “Luigi Vanvitelli”, Via Luciano Armanni 5, 80138 Naples, Italy; 3 Service of Maternal and Child Health, Local Health Unit Napoli 1, Strada Comunale del Principe, 13/a, 80145 Naples, Italy; 4 Department of Gynecology and Obstetrics, Local Health Unit Toscana Centro, Santo Stefano Hospital, 59100 Prato, Italy

**Keywords:** attitudes, COVID-19, knowledge, midwives, pregnant, recommendation, vaccination

## Abstract

This cross-sectional survey investigated the knowledge, attitudes, and practices concerning the COVID-19 vaccination for pregnant women among midwives in Italy and the associated factors. Midwives with at least five years of midwifery education and who had received information about the COVID-19 vaccination from official government organizations or scientific journals were more likely to know in which trimester this vaccine can be administered. A higher perceived utility of this vaccination was observed among midwives working in the public sector, in those concerned by being infected by SARS-CoV-2, who have received at least one dose of this vaccination, in those who considered COVID-19 a severe disease for pregnant women and their fetus, and who believed that the vaccination is safe. One-third of the midwives routinely provided information and half recommended this vaccination. Midwives with more years of activity, who received information about the vaccination from official government organizations or scientific journals, those who had never assisted patients with SARS-CoV-2, and those who believed in midwives’ role in COVID-19 prevention were more likely to routinely provide information. Participants who perceived a higher utility of this vaccination, those who believed in midwives’ role in COVID-19 prevention, those who received information from official government organizations or scientific journals were more likely to routinely provide a recommendation for the vaccine. Midwives’ knowledge must be improved for ensuring that they communicate and recommend the vaccination to their patients.

## 1. Introduction

Once infected with SARS-CoV-2, pregnant women show an increased risk of maternal mortality, caesarean delivery, and preterm birth when compared with pregnant women without COVID-19 [1,2], and of hospitalization, critical care admission, and invasive ventilation than non-pregnant women with COVID-19 [3,4].

It is well-known that the prevention of the SARS-CoV-2 infection through vaccination is the most efficacious measure, along with hand washing with soap and water, wearing face masks, and social distancing. In Italy, the COVID-19 immunization program commenced in December 2020 for the most vulnerable groups; afterwards, the health authorities recommended the vaccination to pregnant women in any trimester and, since September 2021, only during the second and third trimester [5]. Although none of the initial vaccine trials enrolled pregnant women, now there is extensive real-world evidence confirming the vaccine’s safety [6,7,8,9] and effectiveness in pregnancy [10,11,12]. However, the vaccination coverage rate remains unsatisfactory in most countries among this group [13,14,15,16], as for other recommended vaccines [17,18,19], and considerably lower than in the general female population [16], providing arguments to reinforce the recommendations for the reduction in the overall COVID-19 severity and mortality.

Midwives are, among the health care workers (HCWs), those that a pregnant women will see most often and provide active evidence-based health information and promote recommendations about COVID-19 vaccine. Despite this important and crucial role, surprisingly, the literature on attitudes and behaviors towards the COVID-19 vaccination among midwives is very limited [20]. In this context, as it is essential to acquire this information, the first aim of the present survey carried out among a sample of midwives in Italy was to characterize the knowledge, attitudes, and behaviors toward the recommendation of the COVID-19 vaccination to the target population of pregnant women. The second aim was to assess the predictive factors influencing these knowledge, attitudes, and behaviors.

## 2. Materials and Methods

### 2.1. Setting and Participants

The cross-sectional survey used an anonymous web-based survey platform (Lime Survey) and took place between August and November 2022. The study population comprised all 709 midwives who were members of the Italian Society for Obstetrics-Neonatal-Gynecological research (SIRONG).

### 2.2. Procedures

Initially, the research team delivered a letter to the President of the Society with a description of the survey and asking for their permission to conduct it among their members and to obtain their e-mail-addresses. Once the research team had obtained the permission, with the assistance of the Society, all of the members received an e-mail invitation to participate in the online survey. The e-mail contained an information sheet with the lead investigator’s names and contact details, the purposes and methodology of the survey, the importance of their voluntary participation, that the survey could be answered without responding to all questions, that responses were completely anonymous and confidential, and that they had the right to refuse or withdraw their participation at any time without disclosing a reason. The e-mail included a unique link to each participant and directed them to the electronic survey delivered using the platform. Responders gave their informed consent by simply answering the questionnaire and they were only allowed to respond once. To improve the response rate, up to 10 follow-up reminders were sent to non-participants via e-mail at two weekly intervals. The research team did not have direct contact with the participants. No incentives were given to those who participated in the survey.

This survey was reviewed and approved by the Ethics Committee of the Teaching Hospital of the University of Campania “Luigi Vanvitelli” (code 0031987/i).

### 2.3. Survey Instrument

The survey instrument was adapted from questionnaires used in previous studies on this topic carried out by this research group [21,22,23,24,25,26]. The questionnaire was piloted on a sample of 10 non-selected midwives to verify the comprehension of the questions and ease of completion. The results of the pilot survey were not included in the analysis.

Approximately 10 min was required to complete the questionnaire, which was composed by 25 items, structured in five sections. The first section gathered the socio-demographic, professional, and anamnesis data, such as gender, age, marital status, the duration of employment, the setting of their working activity, whether they have a chronic medical condition, whether they have been vaccinated against COVID-19, whether they have been infected with SARS-CoV-2, and whether they know people infected with SARS-CoV-2. The second section assessed the participants’ COVID-19-related knowledge regarding the recommendation of the vaccination for pregnant women, with response options of “yes”, “no”, or “do not know”. The third section determined the participants’ health beliefs toward the SARS-CoV-2 infection and COVID-19 vaccination for pregnant women with 8 questions regarding the level of concern of being infected by SARS-CoV-2, the belief that COVID-19 is a severe disease for pregnant women, their concern about the gravity of the SARS-CoV-2 infection and the safety of the COVID-19 vaccine for pregnant women and their fetus, their perceived utility to receive the COVID-19 vaccination, and their belief that they have an important role in the prevention of COVID-19. The responses were measured using a 10-point Likert scale ranging between 1 = not at all and 10 = at all. The fourth section assessed the practices regarding the frequency of providing information and recommendation to their pregnant patients, measured on a 5-point Likert scale ranging between “never” and “always”. The participants were also asked to provide the reason(s) for the recommendation by using a multiple choice closed-ended question that allowed the respondents to choose one or more answers from a given list of options. The last section asked for the sources from which the participants had obtained information regarding the COVID-19 vaccination for pregnant women by using a multiple choice closed-ended question, which allowed the respondents to choose one or more answers from a given list of options and whether they needed additional information.

### 2.4. Statistical Analysis

Data analysis was conducted using the STATA statistical software version 15.1 [27]. First, the data were analyzed using descriptive statistics, including frequencies, means, and standard deviations to summarize the respondents’ characteristics and their responses. Second, a series of univariate analyses were performed by using a chi-square test or Student’s *t*-test to assess the strength of association, respectively, between the categorical and continuous variables with the different outcomes of interest. All of the independent variables with a *p*-value less than or equal to 0.25 in the univariate analysis were included in the multivariate linear and logistic regression models to determine the factors that are independently associated with each of the dependent variables. Four multivariate models were employed to identify the independent associations between the independent variables and the following outcomes of interest: knowledge that the COVID-19 vaccination is recommended in the second and third trimester of gestation (no = 0; yes = 1) (Model 1); perceived utility to receive the COVID-19 vaccination during pregnancy (continuous) (Model 2); routinely provide information about the COVID-19 vaccination to pregnant women (no = 0; yes = 1) (Model 3); and routinely provide the recommendation of the COVID-19 vaccination to pregnant women (no = 0; yes = 1) (Model 4). The following independent variables were included in all of the models because they are potentially related to all outcomes: gender (other = 0; female = 1); age, in years (continuous); level of midwifery education, in years (less than five = 0; at least five = 1); working in the public setting (no = 0; yes = 1); length of working activity, in years (continuous); having at least a chronic medical condition (no = 0; yes = 1); having received at least one dose of the COVID-19 vaccination (no = 0; yes = 1); having been infected by SARS-CoV-2 (no = 0; yes = 1); having assisted pregnant women infected by SARS-CoV-2 (no = 0; yes = 1); having received information about the COVID-19 vaccination for pregnant women from official government organizations or scientific journals (no = 0; yes = 1); and the need of additional information about the COVID-19 vaccination for pregnant women (no = 0; yes = 1). Moreover, the following variables were included in Models 2–4: knowing in which trimester of gestation the COVID-19 vaccination is recommended (no = 0; yes = 1); concern of being infected by SARS-CoV-2 (continuous); considering the SARS-CoV-2 infection very dangerous for pregnant women and their fetus (no = 0; yes = 1); considering the COVID-19 vaccination safe for pregnant women and their fetus (no = 0; yes = 1); perceived utility of the COVID-19 vaccination during pregnancy (continuous); belief that COVID-19 is a severe disease for pregnant women (continuous); and the belief that midwives have an important role in the prevention of COVID-19 for pregnant women (continuous). The *p* = 0.2 and *p* = 0.4 were used, respectively, to retain or to exclude the variables in the final multivariate models. The Odds Ratios (OR) and their respective 95% confidence interval (CI) were reported in the multivariate logistic regression models, whereas in the linear regression model, the standardized regression coefficient (ß) was used. All of the tests were two-tailed and *p*-values equal to or less than 0.05 were considered to be statistically significant.

## 3. Results

Among the 709 midwives, a total of 260 agreed to participate, with a response rate of 36.7%. A detailed description of the principal socio-demographic, professional, and anamnesis characteristics of the study group are presented in Table 1. The majority of participants were female (96.1%), the mean age was 40.3 years, two-thirds were married or cohabited with a partner, 92.6% were working in the public setting, the mean length of working activity was 14.9 years, only 1.5% had not received the COVID-19 vaccination, 18.9% had at least one chronic medical condition, 71.9% had been infected by SARS-CoV-2 and three-quarters of them were infected after the third dose of the COVID-19 vaccine.

The majority of the midwives knew that the COVID-19 vaccine is recommended for pregnant women (96.8%), although less than one-third of them (32.1%) were aware that it is recommended in the second or third trimester of gestation. The multivariate linear and logistic regression analyses identified several exploratory variables significantly associated with the different outcomes of interest, and the results are reported in Table 2. The midwives who knew the trimesters of gestation in which the COVID-19 vaccine is recommended were those with at least five years of midwifery education (OR = 2.13; 95% CI 1.07–4.23) and those who had received information about the COVID-19 vaccine for pregnant women from official government organizations or scientific journals (OR = 2.02; 95% CI 1.06–3.84) (Model 1).

The midwives expressed a low concern of being infected by SARS-CoV-2, measured on a 10-point Likert scale with an average value of 4.9. The respondents’ belief that COVID-19 is a severe disease for pregnant women, measured on a 10-point Likert scale, resulted in a mean value of 6.3, with only 10.3% giving a value of 10. Only 7.8% of the participants considered the SARS-CoV-2 infection very dangerous for pregnant women and their fetus. The perceived utility of the COVID-19 vaccination during pregnancy was generally high, with a mean value of 7.8 on a 10-point Likert scale, and 40.1% exhibited the highest score, although only one-fourth (24%) considered the vaccination safe for pregnant women and their fetus. More than half of the respondents (53.3%) believed that they had an important role in the prevention of COVID-19 among pregnant women, by responding with the highest score on a 10-point Likert scale, with an overall mean value of 8.2. The multivariate linear regression analysis showed that a higher level of perception regarding the utility of the COVID-19 vaccination during pregnancy was more likely to be observed in midwives working in the public setting, in those with a higher level of concern of being infected by SARS-CoV-2, those who have received at least one dose of the COVID-19 vaccination, those who believed that COVID-19 is a severe disease for pregnant women, and those who considered the vaccination safe for pregnant women and their fetus (Model 2 in Table 2).

Only 36.6% of the sample routinely provided information about the COVID-19 vaccination to the pregnant women, while 4.2% claimed to have never provided this information to pregnant patients. Four variables were found to be significantly associated with the probability of midwives routinely providing information about the COVID-19 vaccination to their patients. The respondents with more years of activity (OR = 1.03; 95% CI 1.01–1.05), those who had received information about the COVID-19 vaccination for pregnant women from official government organizations or scientific journals (OR = 2.57; 95% CI 1.32–5.02), those who have never provided assistance to a pregnant woman infected by SARS-CoV-2 (OR = 0.43; 95% CI 0.23–0.81), and those who believed that midwives have an important role in the COVID-19 prevention for pregnant women (OR = 1.27; 95% CI 1.07–1.5) were more likely to routinely provide information about the COVID-19 vaccination to pregnant women (Model 3 in Table 2).

More than half of the participants routinely recommended the COVID-19 vaccination to their patients (51.9%) and the most common reasons were that, for pregnant women, this vaccination was safe (61.5%), useful (51.6%), safe for the fetus (43.5%), and that COVID-19 is a serious disease for pregnant women (32.8%) and for their fetus (22.1%). Only 5.4% of the participants had never recommended this vaccination and the main reasons were that they believe that COVID-19 is not a serious disease for pregnant women (30.8%), that this vaccination is not safe for them (30.8%) or for the fetus (23.1%), and that it is not useful (23.1%). The results of the multivariate logistic regression model showed that the midwives who perceived a higher utility of the COVID-19 vaccination during pregnancy (OR = 1.45; 95% CI 1.24–1.69), those who believed that midwives have an important role in COVID-19 prevention for pregnant women (OR = 1.25; 95% CI 1.07–1.46), and those who had received information about the COVID-19 vaccination for pregnant women from official government organizations or scientific journals (OR = 2.08; 95% CI 1.12–3.88) were more likely to routinely recommend this vaccination to pregnant women (Model 4 in Table 2).

Almost all of the respondents had acquired information on the COVID-19 vaccination for pregnant women (99.6%). Among the different sources that have been used to acquire knowledge, the most reliable ones were official government organizations (52.8%), scientific societies (35.7%), scientific journals (26.4%), scientific meetings (18.7%), and physicians (13.2%). Almost two-thirds (62.5%) needed further information relating to the COVID-19 vaccination for pregnant women.

## 4. Discussion

To the best of our knowledge, the evaluation of a midwife’s population regarding their knowledge, perceptions, and behaviors towards the COVID-19 vaccination in pregnant women has not been reported previously in Italy, as well as the assessment of the associated factors. Such information is relevant because midwives are among the first to encounter pregnant women and useful findings from this survey include the following.

First, almost all of the midwives were aware of the COVID-19 vaccine recommendation for pregnant women, but only less than one-third knew in which trimester. This inadequate level of knowledge is worrying given the national recommendations and the essential role played by midwives in informing and recommending vaccinations to pregnant women. A higher level of knowledge has been observed about other vaccinations for pregnant women among midwives [28,29] and for their patients among other HCWs [30,31] and is also associated with midwives’ acceptance of the COVID-19 vaccine [32]. Thus, interventions are needed to improve midwives’ knowledge. Such efforts may be even more important in the current situation, given the extraordinary impact of this disease worldwide.

Second, the respondents had a positive attitude towards the utility of the COVID-19 vaccine for pregnant women with a mean value of 7.8 on a 10-point Likert scale, and 40.1% exhibited the highest score. Whilst this attitude could be encouraging, only 7.8% and 24% of the sample, respectively, considered the SARS-CoV-2 infection very dangerous, and the COVID-19 vaccination safe, for pregnant women and their fetus. The results of the multivariate linear regression model identified working in the public setting as a facilitator of considering the COVID-19 vaccine useful for pregnant women. This finding may be explained considering that those working in private practice are more likely to have not received the COVID-19 vaccination, probably because they are more independent and have less contact with their direct superior and, therefore, they may not have easy access to correct and updated information [33]. Moreover, having a higher level of perception regarding the severity of COVID-19 had a positive influence on the perceived utility about the COVID-19 vaccination, which has been previously reported among HCWs regarding the second booster dose [21].

Third, the survey indicated that only 36.6% of the respondents routinely provided information about the COVID-19 vaccination and only 51.9% routinely provided vaccine recommendation to their patients. Thus, this finding is concerning as midwives miss the opportunity to provide this vaccination. This value was higher than the 37.5% in a sample of midwives in France [20]. As indicated in other surveys, a HCW’s recommendation has been shown to be one of the main factors influencing individual’s perceptions and practices about vaccinations [14,34,35,36] and the lack of a recommendation is often a reason for not being immunized [14,37,38,39]. Understanding the barriers is necessary for developing interventions that could increase the vaccination coverage. Among the participants that did not recommend the vaccination, the main reasons were the belief that COVID-19 is not a serious disease for pregnant women and that the vaccine is not safe for them. These finding are unexpected and disappointing as the severity of COVID-19 is well proven [1,2,3,4] and there is a large amount of data supporting the benefits and safety of this vaccination [6,7,8,9,10,11,12]. However, similar concerns have been reported in other studies among pregnant women [14,34,35,36,40] and midwives [41]. Thus, the COVID-19 vaccination coverage among pregnant women may be enhanced by education interventions targeted to midwives to improve their awareness of the clinical burden of the disease and to address their concerns about this vaccination. Moreover, midwives with more years of activity and those who have never provided assistance to a pregnant infected by SARS-CoV-2 had a positive influence in providing information regarding the vaccination. This last result is unexpected, as studies among other groups found that those who knew someone infected were more willing to receive the COVID-19 vaccination [14,26,42]. Furthermore, midwives who considered the COVID-19 vaccination useful for pregnant women were more likely to always recommend it. This is corroborated by previous studies showing that the HCWs who were more confident regarding the benefits and safety of vaccines were more willing to make the recommendation to their patients [31,43,44,45]. Pregnant women should also be informed about the importance of vaccinations and should be encouraged to ask their practitioner about vaccinations.

Fourth, the multivariate logistic regression analyses also revealed that having received information on the COVID-19 vaccination for pregnant women from official government organizations or scientific journals have a significant positive influence. Indeed, midwives who had received information from these sources were more knowledgeable. This result is confirmed by previous studies among HCWs [46,47]. Moreover, having used these sources had a significant influence in providing information and recommending the COVID-19 vaccination. Along with previous research, these findings suggest that midwives, as is the case with other HCWs, should use scientific sources to adopt appropriate practices [48]. By intensifying educational and informative messages, particularly through these sources, higher vaccination coverage should be achieved. Previous studies confirm that these sources are key in improving the knowledge and behaviors of HCWs in terms of recommending these vaccines to their patients [46,48]. Furthermore, it is interesting to observe that almost two-thirds of the participants referred to a need for additional information. This is crucial in order to guarantee that midwives advise and encourage their patients to obtain the COVID-19 vaccination.

As is the case in all similar investigations, the present survey has some potential methodological limitations that should be taken into account when interpreting the results. First, the cross-sectional nature of the survey provides evidence for associations between the independent variables and the outcomes of interest; however, this limits the possibility to establish actual cause-and-effect relationships. Second, the recruitment was conducted online with the possibility of participation bias as the responders may be those with more frequent access to the Internet. This potential bias was managed by sending several survey reminders. Third, the data were collected with a self-administered questionnaire which could subject midwives to response and social desirability bias regarding their attitudes and practices, with either under-reporting or over-reporting despite the assurance of anonymity and confidentiality. Fourth, midwives with a more positive attitude regarding the COVID-19 vaccination and recommendation may be more likely to participate in comparison to those who did not share the same thoughts. This could lead to an overestimation of the number of midwives who recommend the COVID-19 vaccination. Fifth, the survey was conducted among the members of a scientific society and the results might not be generalizable to the whole population of Italian midwives. However, given the attention on this topic among the health care community, we are confident that the sample reflects the likely results of midwives. Finally, the response rate of 36.7% was lower than desired, although similar to other surveys among HCWs [29,48]. Despite the above mentioned limitations, they do not invalidate the findings.

## 5. Conclusions

In conclusion, the findings provide essential information and underline the fact that future efforts are needed to improve midwives’ level of knowledge regarding the COVID-19 vaccination for pregnant women and to support them through health policy in order to have the correct information for ensuring that they can recommend the vaccination to their patients and communicate with them helpfully for increasing uptake rates.

## Figures and Tables

**Table 1:** Main socio-demographic, professional, and anamnesis characteristics of the sample.

Characteristics	N	%
Age, years	40.3 ± 12.6 (20–73) *	
Gender		
Female	250	96.1
Other	10	3.9
Marital status		
Married/cohabited with a partner	169	65.5
Unmarried/separated/divorced/widowed	89	34.5
Level of midwifery education, in years		
Less than five	207	79.6
At least five	53	20.4
Type of work setting		
Public	238	92.6
Private	19	7.4
Length of working activity, in years	14.9 ± 12.5 (1–50) *	
Having been vaccinated against COVID-19		
No	4	1.5
Yes	256	98.5
Having at least one chronic medical condition		
No	211	81.1
Yes	49	18.9
Having been infected by SARS-CoV-2		
No	73	28.1
Yes	187 ^+^	71.9
Before the vaccination	26	13.9
After the first dose of vaccination	7	3.7
After the second dose of vaccination	21	11.2
After the third dose of vaccination	143	76.5
Having assisted pregnant women infected by SARS-CoV-2		
No	80	30.8
Yes	180	69.2

* Mean ± Standard deviation (range). ^+^ 22 of them had been infected twice by SARS-CoV-2. Number for each item may not add up to total number of the study population due to missing value.

**Table 2:** Results of the multivariate logistic and linear regression analysis showing determinants of the different outcomes of interest.

Variable	OR	SE	95% CI	*p*
**Model 1**. Knowledge that the COVID-19 vaccination is recommended in the second and third trimester of gestation Log likelihood = −133.92, χ^2^ = 15.28 (4 df), *p* = 0.0042				
At least five years of midwifery education	2.13	0.74	1.07–4.23	0.03
Having received information about the COVID-19 vaccination for pregnant women from official government organizations or scientific journals	2.02	0.66	1.06–3.84	0.032
Having been infected by SARS-CoV-2	1.88	0.67	0.93–3.79	0.076
Younger	0.98	0.01	0.96–1.01	0.374
	**ß coeff.**	**SE**	***t***	***p***
**Model 2**. Perceived utility to receive the COVID-19 vaccination during pregnancy F(8, 223) = 29.28, *p* < 0.0001, R^2^ = 51.2%, adjusted R^2^ = 49.5%				
Having received at least one dose of the COVID-19 vaccination	4.19	0.96	4.35	<0.001
Considering the COVID-19 vaccination safe for pregnant women and their fetus	2.08	0.29	6.93	<0.001
Belief that COVID-19 is a severe disease for pregnant women	0.37	0.06	6.4	<0.001
Working in the public setting	1.32	0.46	2.87	0.005
Higher concern of being infected by SARS-CoV-2	0.11	0.05	2.2	0.029
Younger	−0.02	0.01	−1.6	0.111
Need of additional information about the COVID-19 vaccination for pregnant women	0.36	0.26	1.36	0.175
At least five years of midwifery education	0.36	0.31	1.19	0.237
	**OR**	**SE**	**95% CI**	***p***
**Model 3**. Routinely provide information about the COVID-19 vaccination to pregnant women Log likelihood = −129.24, χ^2^ = 45.86 (6 df), *p* < 0.0001				
Having received information about the COVID-19 vaccination for pregnant women from official government organizations or scientific journals	2.57	0.88	1.32–5.02	0.005
Believing that midwives have an important role in the COVID-19 prevention for pregnant women	1.27	0.11	1.07–1.5	0.007
Not having assisted pregnant women infected by SARS-CoV-2	0.43	0.14	0.23–0.81	0.009
Longer working activity	1.03	0.01	1.01–1.05	0.027
Female	5.75	6.33	0.66–49.81	0.113
Higher perceived utility to receive the COVID-19 vaccination during pregnancy	1.11	0.08	0.96–1.28	0.166
**Model 4**. Routinely provide the recommendation of the COVID-19 vaccination to pregnant women Log likelihood = −127.12, χ^2^ = 71.03 (4 df), *p* < 0.0001				
Higher perceived utility to receive the COVID-19 vaccination during pregnancy	1.45	0.11	1.24–1.69	<0.001
Believing that midwives have an important role in the COVID-19 prevention for pregnant women	1.25	0.1	1.07–1.46	0.005
Having received information about the COVID-19 vaccination for pregnant women from official government organizations or scientific journals	2.08	0.66	1.12–3.88	0.021
Not having assisted pregnant women infected by SARS-CoV-2	0.64	0.22	0.33–1.24	0.19

## Data Availability

The data presented in this study are available on request from the corresponding author.
